# Percutaneous Transhepatic Retrieval of Dysfunctional Plastic Biliary Stents

**DOI:** 10.1155/rrp/9346732

**Published:** 2026-01-09

**Authors:** Milan Sigdel, Chengzhi Zhang, Manoj Sigdel, Mikias Legesse Gebremedhin, Roshan Bhattarai, Xueliang Zhou, Mengyao Song, Kaihao Xu, Dechao Jiao

**Affiliations:** ^1^ Department of Interventional Radiology, The First Affiliated Hospital of Zhengzhou University, Zhengzhou, Henan, China, zzu.edu.cn; ^2^ Manipal Pokhara College of Medical Science, Kaski, Pokhara, Nepal; ^3^ Tribhuvan University, Kirtipur, Bagmati, Nepal, tribhuvan-university.edu.np

**Keywords:** bile duct obstruction, cholangiography, cholangiopancreatography, endoscopic retrograde, stents

## Abstract

**Purpose:**

To evaluate the safety and efficacy of percutaneous transhepatic retrieval of dysfunctional plastic biliary stent (PBS).

**Materials and Methods:**

In this retrospective, single‐center study, clinical and procedural data of 36 patients who underwent percutaneous transhepatic retrieval of dysfunctional PBS between January 2015 and April 2021 were analyzed. The primary outcomes were technical success, clinical success, complications, procedure time, and radiation exposure. The secondary outcomes were biochemical indicators (total bilirubin [TB], direct bilirubin [DB], glutamic transaminase [ALT], and CA‐19‐9) measured before treatment and two weeks posttreatment.

**Results:**

Technical and clinical success were both 100%. Minor complications occurred in 4 cases (11.2%), including 3 hemobilia (Grade 1) and 1 mild cholangitis (Grade 2). The mean procedure time was 39.22 ± 9.34 minutes, with no statistical significance between benign and malignant disease groups ([40.52 ± 9.0] min vs. [38.91 ± 9.5], *p* = 0.68). The mean radiation exposure was 326.13 ± 206.06 mGy, with no statistical significance between benign and malignant groups ([353.64 ± 258.93] mGy vs. [319.49 ± 196.15] mGy, *p* > 0.75). Liver functions parameter improved significantly after 2 weeks (TB: [168.31 ± 53.07] vs. [41.70 ± 7.29] μmol/L, DB: [133.51 ± 46.42] vs. [25.39 ± 7.26] μmol/L, ALT: [94.67 ± 38.06] vs. [47.41 ± 13.69] U/L, and CA‐19‐9: [479.11 ± 160.14] vs. [150.72 ± 105.72] U/mL) (all *p* = 0.01).

**Conclusion:**

This study suggests that percutaneous transhepatic retrieval of dysfunctional PBS appears to be a safe and effective alternative when ERCP is not feasible. However, given the retrospective single‐center design, limited sample size, and short follow‐up, larger multicenter studies with longer observation are needed to validate these findings.

## 1. Introduction

Biliary stents are commonly used to facilitate bile drainage into the digestive tract. They are frequently employed for the palliation of malignant biliary obstruction and in the management of benign conditions, including biliary fistulas, benign biliary strictures, and iatrogenic bile duct lesions resulting from laparoscopic cholecystectomy [1, 2]. In these patients, interventional radiologists often weigh the decision between placing either a plastic biliary stent (PBS) or a self‐expandable metallic stent (SEMS) [3]. The decision generally depends on expertise, resources, the cost of the stent, the type of disease, and the patient’s life expectancy [4]. The patency of PBS is limited, with a median of 35–165 days, and stent dysfunction can occur, leading to complications such as cholangitis or infection [4, 5]. The main causes of stent dysfunction are the accumulation of sludge and bacterial biofilm on the stent surface, tumor ingrowth, and stent migration [6, 7].

The primary approach for dysfunctional PBS is to remove or replace the stent under endoscopic retrograde cholangiopancreatography (ERCP) at most centers. However, ERCP retrieval of PBS may not be possible due to the following: (I) anatomical reasons such as prior surgical biliojejunal anastomosis or duodenal perforation secondary to stent migration [8, 9]; (II) patient factors such as intolerance to anesthesia, as seen in terminally ill patients who are not fit for ERCP; and (III) patient’s subjective rejection to ERCP approach. In such challenging situations, a percutaneous transhepatic approach can serve as an alternative when ERCP retrieval fails. However, most existing studies on this topic are limited to isolated case reports or small case series, and there remains a lack of systematic evidence regarding its safety and efficacy. Therefore, this study aimed to address this gap by retrospectively evaluating the outcomes of percutaneous transhepatic retrieval of dysfunctional PBS in a single‐center cohort.

## 2. Materials and Methods

### 2.1. Study Design and Patient Population

**Figure 1:**
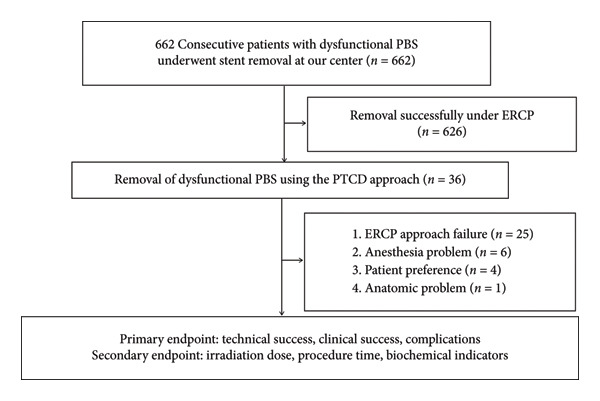
The workflow of the study.

This study received ethical approval from the Research and Clinical Experiment Ethics Committee of The First Affiliated Hospital of Zhengzhou University (2022‐KY‐415; January 15, 2022) and was conducted in accordance with the Declaration of Helsinki (as revised in 2013). Informed consent was obtained from all patients before the procedures. Data were collected from the hospital electronic information system from January 2015 to April 2021. Among 662 patients, 626 patients successfully treated via ERCP were excluded, and clinical data of 36 patients who underwent percutaneous transhepatic retrieval of dysfunctional PBS were retrospectively reviewed. The inclusion criteria were as follows: (I) age 15–90 years, (II) endoscopic approach failure, and (III) high risk associated with airway intubation anesthesia. The exclusion criteria were as follows: (I) severe coagulation dysfunction (PT ≥ 25s, PLT ≤ 30 × 10^9/L), as this increases the risk of bleeding during puncture and retrieval; (II) previous history of recurrent duodenal papilla ulcers, since ulcer‐induced scarring or deformity may distort biliary anatomy and elevate the risk of perforation or bleeding during transhepatic manipulation; and (III) severe ascites, which may obscure visualization and increase the risk of bile leakage or infection during percutaneous access. The study workflow is presented in Figure 1.

### 2.2. Procedure

**Figure 2:**
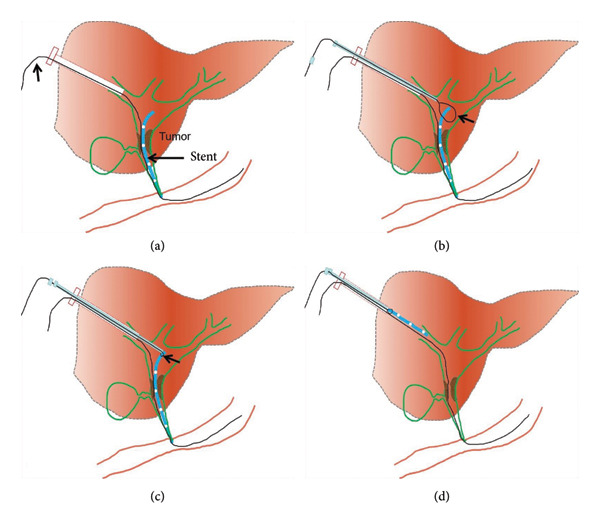
(a): 9F sheath was advanced along the strength guide wire (short arrow) to establish the percutaneous transhepatic access. (b) The 6F gooseneck snare was inserted, and the snare ring (arrow) was opened. (c) Snare ring was adjusted to grasp the proximal segment of the PBS. (d) After fixing the guidewire, the gooseneck snare and dysfunctional PBS were pulled out together.

**Figure 3:**
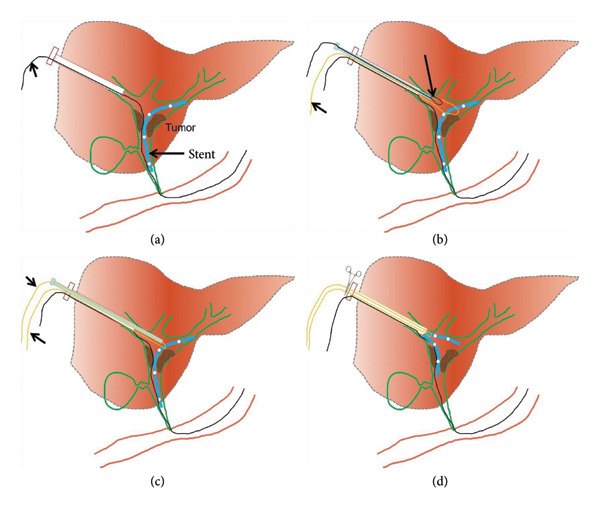
Technique for PBS that had been migrated to the hepatic hilum or the left and right biliary branches. (a) Guidewire‐1 served as the skin–bile duct–duodenum access. (b) The 6F gooseneck snare catheter was inserted through the 9F sheath, and the snare ring was opened. (c) Guidewire‐2 was inserted through the 9F catheter to encircle the middle part of the PBS and fold back into the gooseneck snare ring, and the gooseneck snare was tightened with the 9F sheath’s cooperation. (d) Guidewire‐1 was fixed, and the remaining instruments (Guidewire‐2, gooseneck catcher, and 9F sheath) were pulled out together.

**Figure 4:**
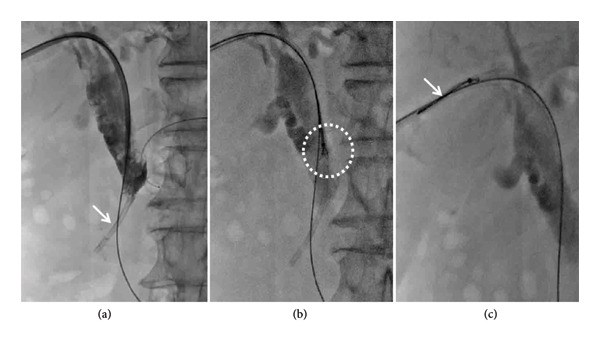
A 78‐year‐old female. (a) Cholangiography showed significant dilation of the bile duct and dysfunctional PBS (white arrow). (b) The 6F gooseneck snare was inserted, and the snare ring was opened and adjusted to grasp the proximal segment of the PBS (white circle). (c) After the Guidewire‐1 was fixed, the gooseneck snare, Guidewire‐2, and dysfunctional PBS were pulled out together.

Percutaneous transhepatic biliary drainage is performed routinely under combined ultrasound and fluoroscopic guidance. Before the procedure, 50 mL of a mixture containing dexmedetomidine hydrochloride (400 μg) and dezocine (20 mg) were intravenously infused (4–12 mL/h) using a pump for pain relief. Ultrasound was used to mark the site of the skin puncture so as to avoid portal or venous branches along the puncture tract. After disinfection, 2% lidocaine (5–10 mL) was used for local anesthesia. A 21G PTC puncture needle (Cook, USA) was used to puncture the intrahepatic segmental dilated biliary branch. Digital subtraction angiography (DSA) (Artis Zeego, Siemens, Germany) was used to visualize the biliary tree and locate the dysfunctional PBS using contrast agent (Hengrui, 300 mg/100 mL, China). Next, a 0.018‐inch platinum guidewire was introduced through the 21G PTC needle, establishing percutaneous access using a 6F sheath (length 15 cm, Cook, USA). A 0.035‐inch guidewire and a 5F KMP catheter (Cook, USA) were maneuvered into the duodenum. This was exchanged for a 0.035‐inch strength guidewire (Terumo, Japan), and a 9F sheath was advanced along the strength guidewire. A 6F gooseneck snare (Hengrui, China) was inserted, and the snare ring was opened and adjusted to grasp the proximal segment of the PBS. After fixing the guidewire, the gooseneck snare and dysfunctional PBS were pulled out together (Figure 2). In case of high biliary drainage where the free end was not accessible, the following technique was used: Two guidewires were used, where Guidewire‐1 served as the skin–bile duct–duodenum access. Then, the 6F gooseneck snare catheter was inserted through the 9F sheath. Guidewire‐2 was inserted through the 9F catheter to encircle the PBS and fold back into the gooseneck snare ring. The gooseneck snare was tightened with the 9F sheath’s cooperation (loop technique), making the PBS securely fixed under fluoroscopy. Guidewire‐1 was fixed, and the remaining instruments (Guidewire‐2, gooseneck catcher, and 9F sheath) were pulled out of the body together (Figures 3 and 4). A dynamic demonstration of the procedure is provided in Supporting Video (Video S1). Forceps biopsy, biliary drainage, or stent implantation (plastic or metal stent) were considered according to the preoperative treatment plans.

### 2.3. Definition

The criteria defining a dysfunctional stent are described in Table 1 according to a previous study [10]. Technical success was defined as the successful retrieval of dysfunctional PBS. Clinical success was defined as a reduction in total bilirubin (TB) by more than 50% within 2 weeks following stent replacement. The white blood cell (WBC) count, alanine aminotransferase (ALT), C‐reactive protein (CRP), TB (TBIL), and carbohydrate antigen‐19‐9 (CA‐19‐9) levels were compared before the procedure and two weeks afterward. Complications were evaluated according to the Society of Interventional Radiology (SIR), Standards of Practice Committee classification on percutaneous hepatobiliary interventions [11]. Minor complications were defined as complications requiring no therapy and with no postprocedure sequel (Grade 1) or complications requiring overnight observation only, with no therapy and no consequence (Grade 2). Major complications were defined as those requiring postprocedure therapy and hospitalization (48 h, Grade 3), causing permanent mild sequels (Grade 4 and Grade 5) and death (Grade 6).

**Table 1:** Stent dysfunction criteria.

Parametric tests		Criteria
Major		New dilatation of intrahepatic or extrahepatic bile ducts on medical imaging

Minor		
1	Bilirubin	≥ 2 mg/dL (34.2 μmol/L) with an increase of ≥ 1 mg/dL (17.1 μmol/L) compared to the value after initial successful drainage
2	Alkaline phosphatases	Twice the upper limit of normal values with an increase of at least 30 U/L
3	Signs of cholangitis	Fever and leukocyte count > 10 000/μL or C‐reactive protein (CRP) > 20 mg/dL

*Note:* Stent dysfunction was defined as the presence of major criteria with any two of the minor criteria.

### 2.4. Statistical Analysis

All statistical analyses were performed using IBM SPSS Statistics 21.0. Quantitative variables with symmetric distribution were described by means and standard deviations and compared by Student’s *t*‐test for independent samples. Paired *t*‐tests were used to compare the same indexes before and after the procedure in the same patient. *p* values <  0.05 were considered statistically significant.

## 3. Results

The study included 36 cases (men/women = 19/17) with a median age of 70 ± 9.9 years (56–88 years). The clinical symptoms were abdominal pain with jaundice (*n* = 24) and jaundice with fever (*n* = 12). Forty‐two dysfunctional PBS (6 cases with two and 30 cases with one dysfunctional PBS each) were removed from the common bile duct. Among them, the loop technique was used for 6 cases with high biliary drainage. All of the retrieved PBS were 6–7F and 60–80 cm long. After dysfunctional PBS retrieval, PBS replacement was performed in 29 cases (69.4%), SEMS placement together with forceps biopsy was performed in 9 cases (25%), and PBS replacement together with forceps biopsy was performed in 2 cases (5.6%). Baseline characteristics, such as age and sex, were compared between the two groups to mitigate potential bias. More information is listed in Table 2.

**Table 2:** Clinical and demographic data of enrolled patients.

Characteristics	Value (mean ± SD, range, and percent)
Total number	36
Age (years), mean ± SD (range)	70.06 ± 9.96 (56–88)
Sex, *n* (%)	
Male	19 (52.8%)
Female	17 (47.2%)
Clinical symptoms, *n* (%)	
Abdominal pain with jaundice	24 (66.7%)
Jaundice with fever	12 (33.3%)
Biliary stenosis reasons	
Malignant stenosis, *n* (%)	29 (80.6%)
Benign stenosis, *n* (%)	7 (19.4%)
Number of dysfunctional PBS	
Single (*n* = 1)	30 (83.3%)
Multiple (*n* ≥ 2)	6 (16.7%)
BMI (kg/m^2^; mean ± SD; range)	24.83 ± 3.07 (19.5–30.1)
Time to treatment (day)	37.19 ± 16.24 (18.0–70.0)
The causes of choosing PTCD approach, *n* (%)	
ERCP approach failure	25 (69.4%)
Anesthesia problem	6 (16.7%)
Patient preference	4 (11.1%)
Anatomic problem	1 (2.8%)
Procedure time (min; mean ± SD; range)	39.22 ± 9.34 (22.2–61.2)
Irradiation dose (mGy; mean ± SD; range)	326.13 ± 206.06 (100.8–942.6)
Technical success (%)	36/36 (100%)
Clinical success (%)	36/36 (100%)
Complications, *n* (%)	
Major complications	0
Minor complications	4 (11.2%)
Following local treatments	
PBS replacement	29 (69.4%)
SEMS placement + forceps biopsy	9 (25%)
PBS replacement + forceps biopsy	2 (5.6%)

Abbreviations: PBS, plastic biliary stent; SEMS, self‐expandable metallic stent.

Both technical success and clinical success rates were 100%. The mean radiation dose was 326.13 ± 206.06 mGy (range: 100.8–942.6 mGy), and further stratified analysis showed no significant difference between benign and malignant biliary obstructions (353.64 ± 258.94 vs. 319.49 ± 196.19 mGy, *p* = 0.70). The mean procedure time was 39.22 ± 9.34 min (range: 22.2–61.2 min) with no significant difference in procedure time between benign and malignant biliary obstructions (40.52 ± 9.00 vs. 39.91 ± 9.55 min, *p* = 0.68). In addition to improvements in liver enzymes (ALT, TBIL, and DBIL), significant reductions in WBC, CRP, and CA‐19‐9 were observed two weeks postprocedure (*p* < 0.001), suggesting effective relief of biliary obstruction, improvement in hepatic function, and resolution of inflammatory response (Table 3). No major complications occurred. Minor complications occurred in four cases (11.2%), including three cases of hemobilia (Grade 1) and one case of mild cholangitis (Grade 2).

**Table 3:** Comparison of biochemical indicators.

Characteristics	Pretreatment	2 weeks later	*p* value
WBC (× 10^12^/L)	13.49 ± 5.4	7.21 ± 2.12	0.01
ALT (U/L)	94.67 ± 38.06	47.41 ± 13.69	0.01
TBIL (μmol/L)	168.31 ± 53.07	41.70 ± 7.29	0.01
DBIL (μmol/L)	133.51 ± 46.42	25.39 ± 7.26	0.01
CRP (mg/L)	60.26 ± 18.84	22.16 ± 8.90	0.01
CA‐19‐9 (U/mL)	479.11 ± 160.14	150.72 ± 105.72	0.01

*Note:* TBIL, total bilirubin; DBIL, direct bilirubin.

Abbreviations: ALT, alanine aminotransferase; CA‐19‐9, carbohydrate antigen‐19‐9; CRP, C‐reactive protein; WBC, white blood cell.

## 4. Discussion

Dysfunctional stents result from sludge, bacterial biofilm deposition, and tumor ingrowth or overgrowth within the lumen, and side holes in the case of PBS or SEMS [12, 13]. While SEMS offers longer stent patency, it is more expensive and involves a relatively complex insertion procedure [14]. Conversely, inserting PBS is cheaper and simpler, but the average patency of PBS is only 35–165 days [15]. Therefore, timely replacement of PBS within 3–6 months using an endoscopic approach is recommended to prevent stent dysfunction [16].

The technique of PBS dislodgement by pushing it into the duodenum has been described for dysfunctional PBS after endoscopic approach failure in various studies either using (I) a long introducer or (II) a balloon catheter inflated coaxially or (III) an over‐the‐wire technique. Fotheringham [17] and Brown et al. [18] reported the use of a balloon dilatation catheter to push the stent into the duodenum. Similarly, Lagana et al. [19], in their study of 8 cases, reported the use of a goose neck snare to push the stent into the duodenum with no major complications. Although the success rate in these studies was 90%–100% and without no any immediate complications, these procedures were described as complex and time‐consuming [16–18]. Furthermore, cases of perforation of the terminal ileum and colon following dislodgement were often reported as complications [17–21]. Regarding retrieval via percutaneous transhepatic approach, there are only a few studies, which are mainly case reports or studies with limited sample sizes (Table 4). Celii and Zvavanjanja [24] and Hsien‐Tzu et al. [23] have reported using forceps to grasp the dysfunctional PBS and retrieve it percutaneously. Gupta et al. [22], in their case report, described the method of using a goose neck snare to remove PBS percutaneously without any major complications. Gumus [25], in his study of 43 dysfunctional PBS, reported 6 failed cases of failure of a balloon catheter and “over‐the‐wire” technique to push the PBS into the duodenum. These cases were subsequently managed successfully with percutaneous transhepatic retrieval using a goose neck catheter.

**Table 4:** Studies on percutaneous transhepatic retrieval of dysfunctional stent.

Year	Author/country	Design	SS	M/F	Mean Age (year)	Methods	TS (%)	Complications
2018 [20]	Francis Celii/USA	CR	1	1/0	70	Grasping with bronchial forceps	100	No complications
2017 [21]	Liu Hsien‐Tzu/China	CR	2	2/0	79.5	1‐Biopsy forceps 1‐Balloon dilatation catheter	100	Not mentioned
2013 [22]	UG. Rossi/Italy	CR	1	0/1	71	Balloon dilatation catheter	100	No complications
2012 [23]	Gümüş B/Turkey	RS	6	—	64.8	Retrieval by snaring	100	Cholangitis
2002 [24]	Gupta A/Australia	CR	1	1/0	54	Retrieval by snaring	100	Minor (pyrexia)

*Note:* M, male; F, female.

Abbreviations: CR, case report; NA, not available; RS, retrospective study; SS, sample size; TS, technical success.

All these methods described in the literature required access to either end of the stent; if both ends were impacted in the biliary tract, snaring or grasping was not possible. In this study, we introduced a loop technique for PBS retrieval for high biliary drainage where either end of the stent is not accessible. By strategically looping the guidewire around the middle segment of the stent and bringing the ends outside, it offered a controlled means of dislodging and extracting the stent from its position within the bile duct (Figure 3). Both the technical success and clinical success rates were 100%. Minor complications occurred in 4 cases (11.2%), which is consistent with previous percutaneous biliary interventions [26–29].

Recent studies have emphasized the expanding role of interventional radiology in complex biliary management. The percutaneous “Y” stent‐in‐stent technique reported by Corvino et al. [30] demonstrated high technical success in advanced malignant hilar strictures, underscoring the versatility and safety of the transhepatic approach in the placement of biliary stents. In our study, although the percutaneous transhepatic technique demonstrated a 100% technical and clinical success rate, this did not include a direct comparison with other percutaneous retrieval methods such as balloon dislodgement or over‐the‐wire techniques. Therefore, the advantages described are based on procedural experience rather than comparative data. Despite theoretical risks that might be associated with bending and pulling the stent in a loop technique, findings indicated minimal liver and bile duct damage from the loop technique. This might be due to the following: (I) smooth stent surfaces from prolonged bile exposure, (II) excellent compliance and deformation of plastic stents, and (III) effective preoperative pain management. Furthermore, the procedures were performed by an experienced interventional radiologist, and preprocedural imaging review and selection of significantly dilated segmental intrahepatic bile duct mitigated potential risks.

Regarding improving the efficacy of percutaneous transhepatic retrieval of PBS, the experience at our center was as follows: (I) Before the procedure, the patient’s enhanced CT or MRI data should be carefully reviewed, and the best intrahepatic bile should be selected. The best intrahepatic bile duct for puncture access should have the following characteristics: (a) No portal or venous branches along the puncture route, which can be assured by using ultrasound guidance. (b) Significant dilatation (c) close to the skin puncture site. (II) The angle between the puncture and the perihepatic dilated bile duct should be greater than 90 degrees in order to facilitate the smooth entry of the 9F sheath into the bile duct. (III) For febrile patients, percutaneous biliary drainage should be performed first. The retrieval of the PBS should be attempted only after the inflammatory indicators have decreased. This approach not only decreases the procedural duration but also reduces the risk of bilirubinemia caused by repeated injection of contrast agents. (IV) For low bile duct drainage, the upper part of the PBS can be directly grasped using a gooseneck catcher. For high bile duct drainage where the proximal part of the PBS is not accessible, a loop technique can be established, encircling the middle of the stent to remove the stent. (V) For patients with poor response to ongoing antitumor treatment, bile duct biopsy can be completed simultaneously after PBS retrieval, providing a clinical basis for further adjustment of treatment plans.

Our study has several limitations. First, it was a retrospective, single‐center study with a relatively small number of patients, which may introduce selection bias and limit the generalizability of the results. Second, the follow‐up period was short (2 weeks), preventing the assessment of long‐term outcomes or delayed complications. Third, a detailed subgroup analysis (e.g., single versus multiple stents or benign versus malignant etiologies) was not performed, as the study was primarily designed to evaluate the overall feasibility and safety of the percutaneous retrieval technique rather than comparative outcomes.

In conclusion, the percutaneous transhepatic approach is a feasible, safe, and effective alternative for PBS retrieval. However, it should be reserved for situations where ERCP is not feasible or has failed. Further prospective, multicenter studies with longer follow‐up are essential to establish its long‐term safety, efficacy, and role in routine clinical practice.

## Ethics Statement

This single‐center study was approved by the Ethics Review Committee of The First Affiliated Hospital of Zhengzhou University (2022‐KY‐415) and conducted in accordance with the Declaration of Helsinki (revised 2013). The requirement for informed consent was waived due to its retrospective nature.

The authors are accountable for all aspects of the work in ensuring that questions related to the accuracy or integrity of any part of the work are appropriately investigated and resolved.

## Consent

Please see the Ethics Statement.

## Disclosure

All authors approved the final manuscript for publication. All authors have completed the ICMJE uniform disclosure form.

## Conflicts of Interest

The authors declare no conflicts of interest.

## Author Contributions

Conception and design: Milan Sigdel. Administrative support: Dechao Jiao. Provision of study materials or patients: Dechao Jiao. Collection and assembly of data: Milan Sigdel and Chengzhi Zhang. Data analysis and interpretation: Milan Sigdel, Manoj Sigdel, and Mikias Legesse Gebremedhin. Manuscript writing: all authors.

## Funding

No specific funding was obtained for this study.

## Supporting Information

Video S1: A dynamic demonstration of the retrieval of a biliary stent by the loop procedure under fluoroscopy.

## Supporting information


**Supporting Information** Additional supporting information can be found online in the Supporting Information section.

## Data Availability

The datasets used and analyzed in this study are available from the corresponding author upon reasonable request.

## References

[1] Dumonceau J. M. , Heresbach D. , Deviere J. , Costamagna G. , Beilenhoff U. , and Riphaus A. , European Society of Gastrointestinal E: Bliary Stents: Models and Methods for Endoscopic stenting, Endoscopy. (2011) 43, no. 7, 617–626, 10.1055/s-0030-1256315, 2-s2.0-79959843619. 21614754

[2] Gwon D. I. , Ko G. Y. , Sung K. B. et al., Clinical Outcomes After Percutaneous Biliary Interventions in Patients With Malignant Biliary Obstruction Caused by Metastatic Gastric Cancer, Acta Radiologica. (2012) 53, no. 4, 422–429, 10.1258/ar.2012.110703, 2-s2.0-84860649065. 22403081

[3] Soehendra N. and Reynders-Frederix V. , Palliative Bile Duct Drainage: A New Endoscopic Method of Introducing a Transpapillary Drain, Endoscopy. (1980) 12, no. 1, 8–11, 10.1055/s-2007-1021702, 2-s2.0-0018872470. 7353562

[4] Wilcox C. M. , Kim H. , Seay T. , and Varadarajulu S. , Choice of Plastic or Metal Stent for Patients With Jaundice With Pancreaticobiliary Malignancy Using Simple Clinical Tools: A Prospective Evaluation, BMJ Open Gastroenterol. (2015) 2, no. 1, 10.1136/bmjgast-2014-000014, 2-s2.0-84969428377. PMC4599157 26462270

[5] Farani M. , Saldi S. R. F. , Maulahela H. , Abdullah M. , Syam A. F. , and Makmum D. , Survival, Stent Patency, and Cost-Effectiveness of Plastic Biliary Stent Versus Metal Biliary Stent for Palliation in Malignant Biliary Obstruction in a Developing Country Tertiary Hospital, JGH Open. (2021) 5, no. 8, 959–965, 10.1002/jgh3.12618. 34386606 PMC8341186

[6] Cacaci M. , De Maio F. , Matteo M. V. et al., Pilot Study on Cultural and Metagenomic Analysis of Bile and Biliary Stentslead to Unveiling the Key Players in Stent Occlusion, Scientific Reports. (2024) 14, no. 1, 10.1038/s41598-024-51480-2. PMC10858256 38336904

[7] Jiao D. , Wu G. , Ren J. , and Han X. , Study of Self-Expandable Metallic Stent Placement Intraluminal (125)I Seed Strands Brachytherapy of Malignant Biliary Obstruction, Surgical Endoscopy. (2017) 31, no. 12, 4996–5005, 10.1007/s00464-017-5481-5, 2-s2.0-85021159667. 28643064

[8] Trejo-Avila M. , Valenzuela-Salazar C. , and Herrera-Esquivel J. J. , Biliary Stent-Induced Duodenal Perforation, Revista de Gastroenterología de México. (2020) 85, no. 3, 358–359, 10.1016/j.rgmxen.2020.01.002. 32336593

[9] Schumacher B. , Othman T. , Jansen M. , Preiss C. , and Neuhaus H. , Long-Term Follow-Up of Percutaneous Transhepatic Therapy (PTT) in Patients With Definite Benign Anastomotic Strictures After Hepaticojejunostomy, Endoscopy. (2001) 33, no. 5, 409–415, 10.1055/s-2001-14264, 2-s2.0-0034961517. 11396758

[10] Schmidt A. , Riecken B. , Rische S. et al., Wing-Shaped Plastic Stents vs. Self-Expandable Metal Stents for Palliative Drainage of Malignant Distal Biliary Obstruction: A Randomized Multicenter Study, Endoscopy. (2015) 47, no. 5, 430–436, 10.1055/s-0034-1391232, 2-s2.0-84928560440. 25590188

[11] Filippiadis D. K. , Binkert C. , Pellerin O. , Hoffmann R. T. , Krajina A. , and Pereira P. L. , Cirse Quality Assurance Document and Standards for Classification of Complications: The Cirse Classification System, CardioVascular and Interventional Radiology. (2017) 40, no. 8, 1141–1146, 10.1007/s00270-017-1703-4, 2-s2.0-85020197563. 28584945

[12] Moy B. T. and Birk J. W. , An Update to Hepatobiliary Stents, Journal of Clinical and Translational Hepatology. (2015) 3, no. 1, 67–77, 10.14218/jcth.2014.00040. 26357636 PMC4542081

[13] Sigdel M. , Zhou X. , Song M. , Liu Y. , Zhang C. , and Jiao D. , A Novel Technique to Remove Migrated Esophageal Stent Under Fluoroscopy, Abdominal Radiology. (2024) 49, no. 5, 1646–1652, 10.1007/s00261-024-04281-0. 38592493

[14] Choi J. H. , Lee S. H. , You M. S. et al., Step-Wise Endoscopic Approach to Palliative Bilateral Biliary Drainage for Unresectable Advanced Malignant Hilar Obstruction, Scientific Reports. (2019) 9, no. 1, 10.1038/s41598-019-48384-x, 2-s2.0-85072194504. PMC6744501 31519930

[15] Sigdel M. , Zhang C. , Hou R. , Song M. , Sun Z. , and Jiao D. , Biliary Metallic Stent Combined With Radioactive 125I Seeds Strands for Malignant Hilar Obstruction, BMC Cancer. (2025) 25, no. 1, 10.1186/s12885-025-13627-w. PMC11809024 39930335

[16] Di Giorgio P. , Manes G. , Grimaldi E. et al., Endoscopic Plastic Stenting for Bile Duct Stones: Stent Changing on Demand or Every 3 Months: A Prospective Comparison Study, Endoscopy. (2013) 45, no. 12, 1014–1017, 10.1055/s-0033-1344556, 2-s2.0-84889572015. 24288221

[17] Fotheringham T. , Abbass S. , Varghese J. C. , Haslam P. , Lyon S. , and Lee M. J. , Displacement of Occluded Plastic Endoprostheses Into the Duodenum During Percutaneous Biliary Drainage: Description of an Under-Reported Technique, Clinical Radiology. (2002) 57, no. 12, 1113–1117, 10.1053/crad.2002.1115, 2-s2.0-0036901508. 12475537

[18] Brown K. T. , Schubert J. , Covey A. M. , Brody L. A. , Sofocleous C. T. , and Getrajdman G. I. , Displacement of Endoscopically Placed Plastic Biliary Endoprostheses Into the Duodenum With a Simple Transhepatic Technique, Journal of Vascular and Interventional Radiology. (2004) 15, no. 10, 1139–1143, 10.1097/01.rvi.0000136292.23500.0a, 2-s2.0-5144226239. 15466802

[19] Lagana D. , Carrafiello G. , Mangini M. et al., An Innovative Percutaneous Technique for the Removal and Replacement of Dysfunctioning Plastic Biliary Endoprostheses (PBE) in the Management of Malignant Billiary Occlusions, Radiologia Medica, La. (2007) 112, no. 2, 264–271, 10.1007/s11547-007-0140-x, 2-s2.0-33947501651. 17361371

[20] Zorbas K. A. , Ashmeade S. , Lois W. , and Farkas D. T. , Small Bowel Perforation From a Migrated Biliary Stent: A Case Report and Review of Literature, World Journal of Gastrointestinal Endoscopy. (2021) 13, no. 10, 543–554, 10.4253/wjge.v13.i10.543. 34733414 PMC8546564

[21] Storkson R. H. , Edwin B. , Reiertsen O. , Faerden A. E. , Sortland O. , and Rosseland A. R. , Gut Perforation Caused by Biliary Endoprosthesis, Endoscopy. (2000) 32, no. 1, 87–89, 10.1055/s-2000-87, 2-s2.0-0033959355. 10691280

[22] Gupta A. , Frazer C. , and Brennan F. , Percutaneous Retrieval of a Proximally Migrated Common Bile Duct Endoprosthesis From the Right Anterior Duct, Australasian Radiology. (2002) 46, no. 3, 325–328, 10.1046/j.1440-1673.2002.01071.x, 2-s2.0-0036042558. 12196248

[23] Hsien-Tzu L. , Hsiuo Shan T. , Nai Chi C. , Yi Yang L. , Yi You C. , and Chien A. L. , Percutaneous Transhepatic Techniques for Retrieving Fractured and Intrahepatically Dislodged Percutaneous Transhepatic Biliary Drainage Catheters, Diagnostic and Interventional Radiology. (2017) 23, no. 6, 461–464, 10.5152/dir.2017.17064, 2-s2.0-85032958376. 29097348 PMC5669547

[24] Celii F. G. and Zvavanjanja R. C. , Percutaneous Transhepatic Use of Rigid Bronchial Forceps as Bailout in Difficult Biliary Stent Retrieval, Radiology Case Reports. (2019) 14, no. 2, 246–250, 10.1016/j.radcr.2018.10.034, 2-s2.0-85056797641. 30479681 PMC6250801

[25] Gumus B. , Percutaneous Intervention Strategies for the Management of Dysfunctioning Biliary Plastic Endoprostheses in Patients With Malignant Biliary Obstruction, Diagnostic and Interventional Radiology. (2012) 18, no. 5, 503–507, 10.4261/1305-3825.DIR.5219-11.2, 2-s2.0-84866104893. 22477647

[26] Rossi U. G. , Rigamonti P. , and Cariati M. , Malfunctioning Plastic Biliary Endoprosthesis: Percutaneous Transhepatic Balloon Pulling Technique, Case Reports in Radiology. (2013) 2013, 596480–596483, 10.1155/2013/596480. 23984158 PMC3745893

[27] Kokas B. , Szijarto A. , Farkas N. et al., Percutaneous Transhepatic Drainage Is Safe and Effective in Biliary Obstruction: A Single-Center Experience of 599 Patients, PLoS One. (2021) 16, no. 11, 10.1371/journal.pone.0260223. PMC8601527 34793565

[28] Khan R. , Hussain Z. , Bari V. , and Fiaz A. B. , Safety of Percutaneous Transhepatic Biliary Stenting in Patients With Obstructive Jaundice, Journal of College of Physicians and Surgeons Pakistan. (2019) 29, no. 1, 24–28, 10.29271/jcpsp.2019.01.24, 2-s2.0-85059828495. 30630564

[29] Sigdel M. , Fang Y. , Sun Z. , Sigdel M. , and Jiao D. , Robotic Navigation-Assisted Percutaneous Liver Puncture: A Pilot Study, Quantitative Imaging in Medicine and Surgery. (2025) 15, no. 2, 1543–1554, 10.21037/qims-24-1584. 39995746 PMC11847208

[30] Corvino F. , Centore L. , Soreca E. , Corvino A. , Farbo V. , and Bencivenga A. , Percutaneous Y Biliary Stent Placement in Palliative Treatment of Type 4 Malignant Hilar Stricture, Journal of Gastrointestinal Oncology. (2016) 7, no. 2, 255–261, 10.3978/j.issn.2078-6891.2015.069, 2-s2.0-84995810489. 27034794 PMC4783754

